# Supplementary material for: Evaluation of the effect of the application of Quercus cerris extract and the use of fluoride bonding material on the bonding strength of orthodontic brackets after tooth bleaching with hydrogen peroxide
Source: PeerJ. 2025 Apr 29;13:e19335. doi: 10.7717/peerj.19335 (PMC12047214; doi:10.7717/peerj.19335)
Supplement: Supplemental Information 1 [file peerj-13-19335-s001.pdf]

These are my research’s photos.
1.Sodium ascorbat synthetic antioxidant
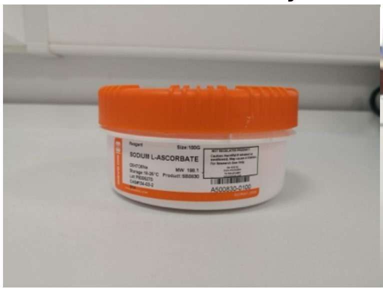

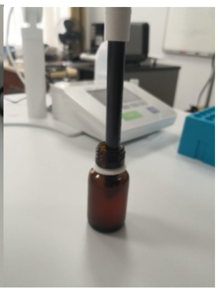
2.The teeth that is the part of my research

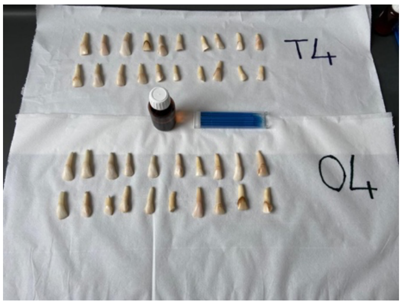

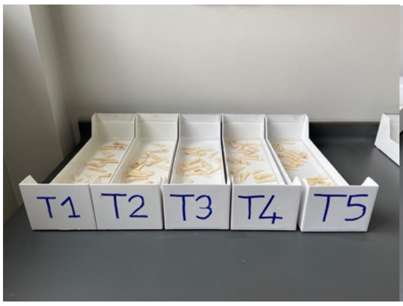

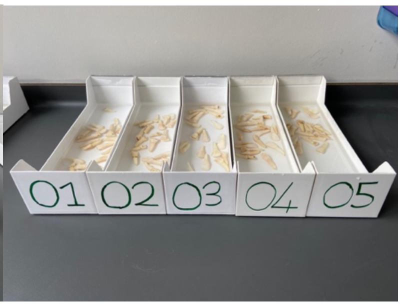

3.The bleached bovine insicors
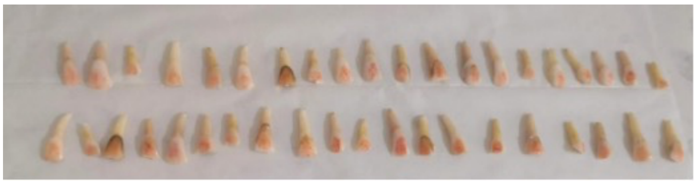
4.The bleaching kit that ı used
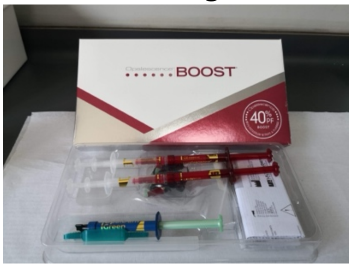

5.The bovine insicors that ı used

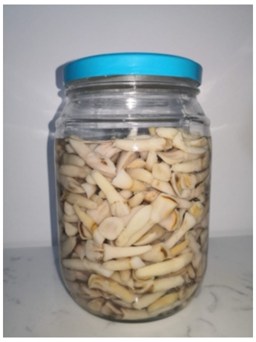
6.The embedded teeth

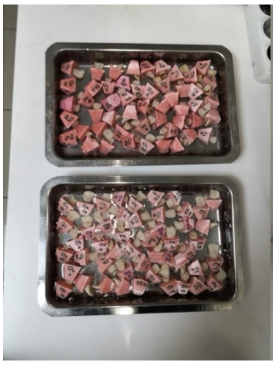
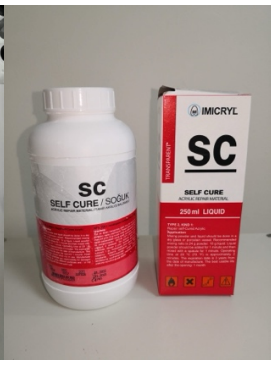

7.This is the ph meter for Q cerris extract

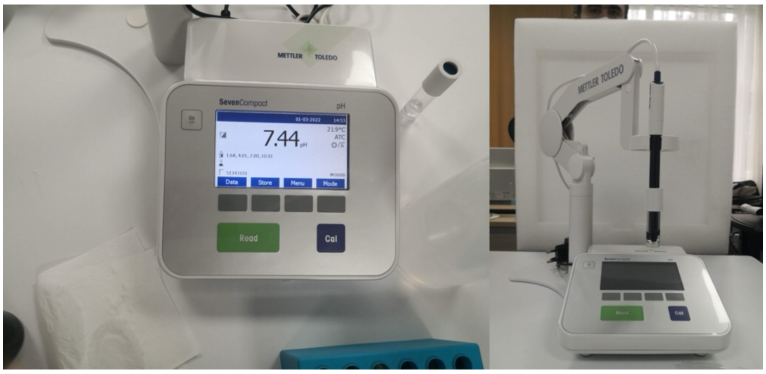

8.The Q cerris extract preparation

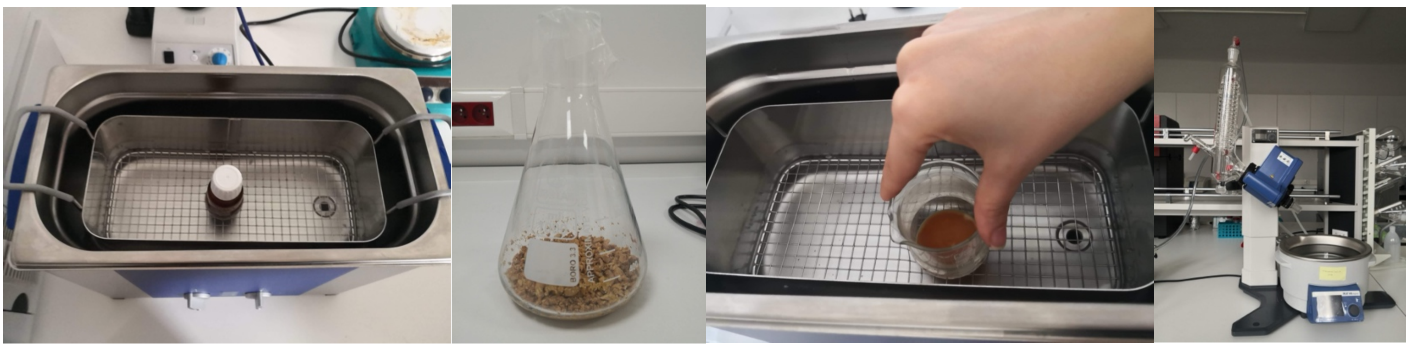

9.The ultrasonic bath

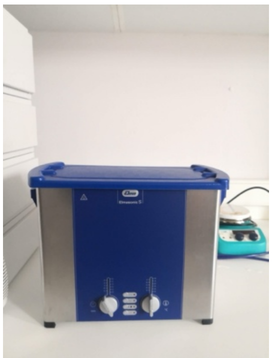

10.The thermocycle machine for teeth

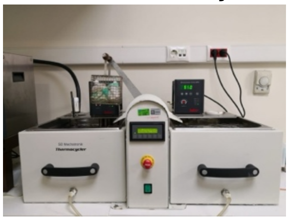

11.Sensitive scale for preparation of sodium ascorbate and Q cerris extract

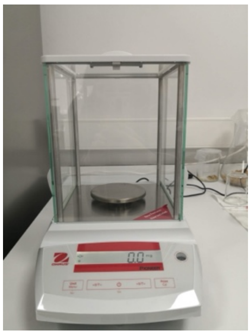

Best Regards..
